# Multiplicative Auditory Spatial Receptive Fields Created by a Hierarchy of Population Codes

**DOI:** 10.1371/journal.pone.0008015

**Published:** 2009-11-24

**Authors:** Brian J. Fischer, Charles H. Anderson, José Luis Peña

**Affiliations:** 1 Department of Mathematics, Occidental College, Los Angeles, California, United States of America; 2 Division of Biology, California Institute of Technology, Pasadena, California, United States of America; 3 Department of Anatomy and Neurobiology, Washington University School of Medicine, St. Louis, Missouri, United States of America; 4 Dominick P. Purpura Department of Neuroscience, Albert Einstein College of Medicine, Bronx, New York, United States of America; Ludwig Maximilians University Munich, Germany

## Abstract

A multiplicative combination of tuning to interaural time difference (ITD) and interaural level difference (ILD) contributes to the generation of spatially selective auditory neurons in the owl's midbrain. Previous analyses of multiplicative responses in the owl have not taken into consideration the frequency-dependence of ITD and ILD cues that occur under natural listening conditions. Here, we present a model for the responses of ITD- and ILD-sensitive neurons in the barn owl's inferior colliculus which satisfies constraints raised by experimental data on frequency convergence, multiplicative interaction of ITD and ILD, and response properties of afferent neurons. We propose that multiplication between ITD- and ILD-dependent signals occurs only within frequency channels and that frequency integration occurs using a linear-threshold mechanism. The model reproduces the experimentally observed nonlinear responses to ITD and ILD in the inferior colliculus, with greater accuracy than previous models. We show that linear-threshold frequency integration allows the system to represent multiple sound sources with natural sound localization cues, whereas multiplicative frequency integration does not. Nonlinear responses in the owl's inferior colliculus can thus be generated using a combination of cellular and network mechanisms, showing that multiple elements of previous theories can be combined in a single system.

## Introduction

The barn owl is able to pinpoint sound sources with great accuracy after hearing only a short burst of sound [1]. This orienting response is mediated by spatially-selective auditory neurons in the midbrain [2], [3]. Spatial selectivity arises in these auditory neurons as a result of computations performed on the auditory input signals (for review see, [4], [5]). Multiplication is believed to be an essential computation in the generation of spatially selective auditory neurons in the owl's midbrain [6], [7].

Space-specific neurons in the barn owl's auditory space map gain spatial selectivity as a result of tuning to combinations of the interaural time difference (ITD) and interaural level difference (ILD; [8], [9]). In the barn owl, as in mammals, ITD is correlated with the horizontal position of a sound source [8], [10]–[12]. A vertical asymmetry of the owl's ears causes ILD to vary primarily with the vertical position of a sound source [10], [11], [13]. Under natural listening conditions, the ITD and ILD at each frequency of the sound stimulus are shaped in a direction-dependent manner [13]–[15]. Therefore, the cues for sound localization consist of ITD and ILD at an array of frequencies. The localization cues ITD and ILD are processed in parallel pathways in the brainstem [16]–[18], where neurons are narrowly tuned to sound frequency (Reviews: [4], [5]). ITD and ILD initially converge in the lateral shell of the central nucleus of the inferior colliculus (ICcl), where neurons remain narrowly tuned to frequency [19]–[21]. The response to ITD and ILD is nonlinear at the site of ITD-ILD convergence in ICcl, but there is a diversity of combination-selective responses over the population [7]. In the next stage, signals converge across frequency in the external nucleus of the inferior colliculus (ICx), where the auditory space map is generated [22], [23]. The response to ITD and ILD in the membrane potential of space-specific neurons in ICx is well described by a multiplication of an ITD-dependent component and an ILD-dependent component [6].

While the owl's auditory system provides one of the best examples of multiplication in a neural circuit, how the sound localization cues are processed under natural listening conditions and what mechanisms produce the responses of neurons in the owl's inferior colliculus remain unanswered questions. Peña and Konishi [6] observed multiplication using sound signals that differ binaurally by a frequency-independent ITD and a frequency-independent ILD. However, under natural listening conditions, the ITD and ILD at each frequency of the sound stimulus are shaped in a direction-dependent manner [13]–[15]. A complete model of sound localization in the owl must thus address how the ITD and ILD tunings are combined with each other and across frequency to generate the responses of space-specific neurons. Previous models of neurons in the owl's midbrain have reproduced aspects of neural responses to sound source direction for single and multiple sound sources [14], [24]–[26]. Common among these models is the assumption that spiking responses of space-specific neurons results from some form of nonlinear interaction between ITD and ILD, both within and across frequency [6], [14], [27], [28]. However, frequency integration in the membrane potential of ICx neurons appears linear [29]. Nor have previous models addressed key aspects of combination selectivity for ITD and ILD observed in the spiking responses of ICcl neurons. In particular, models that explicitly include ICcl assume that combination selectivity for ITD and ILD is uniform over the population [24], [25], whereas experimental measurements reveal a diversity of responses to ITD and ILD in ICcl [7].

Here, we develop a model for the responses of ITD- and ILD-sensitive neurons in the barn owl's inferior colliculus. This work combines multiple experimental data sets [6], [7], [29] to model computational operations that map onto specific regions of the inferior colliculus. We propose that multiplication occurs between ITD and ILD within frequency channels, but that integration across frequency occurs using a linear-threshold operation. This interpretation not only is based on physiological data on the combination of ITD and ILD, but it is also consistent with responses when multiple sound sources are presented [30].

## Methods

### 2.1 Neurophysiology

#### 2.1.1 Ethics statement

Experimental procedures followed the National Institutes of Health *Guide for the Care and Use of Laboratory Animals* and were approved by the Institutional Animal Care and Use Committee of the California Institute of Technology.

#### 2.1.2 Methods

Methods for surgery, stimulus delivery, and data collection have been described previously [6], [7].

### 2.2 Time-Dependent Model of Responses to ITD and ILD in the Inferior Colliculus

We present a model for the responses of ITD- and ILD-sensitive neurons in the barn owl's inferior colliculus. The model is constructed in three stages: (1) a front end for extracting binaural localization cues from the auditory inputs, (2) ICcl, and (3) ICx.

#### 2.2.1 Binaural cue extraction

In the first stage of the model, the input signals to the left and right ears are filtered with a bank of band-pass filters [26]. Each filter is a gamma-tone function with an impulse response given by 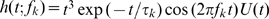, where 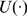 is the unit step function [31]. The center frequency 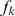 of the filter corresponds to the characteristic frequency of an auditory-nerve fiber. The time constant 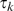 is selected so that the 10 dB width of the gamma-tone filter is equal to the width of the frequency tuning curve of an auditory-nerve fiber with characteristic frequency 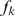, computed 10 dB above threshold [32]. A Gaussian white noise signal is added to the deterministic signal, to model the stochastic representation of signals by populations of neurons.

For an input signal 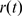, we denote the output of the band-pass filter with center frequency 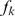 as 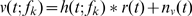 where 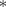 denotes convolution and, for each *t*, 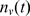 is a Gaussian random variable with mean zero and standard deviation equal to 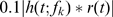. The noise 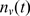 is assumed to have a Gaussian distribution with a standard deviation that scales with the signal because it models the noise in a sum of many neurons' responses, where the variability of each neuron's response increases with the strength of the signal. Noise signals introduced in the sequel are defined similarly and are taken as zero-mean Gaussian random signals, uncorrelated in time, with standard deviation equal to 0.1 times the input signal magnitude. When both the left and right components are considered, the signals are written with subscripts, e.g., 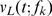 and 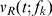.

ITD is extracted from the outputs of the band-pass filterbank using a cross-correlation-based operation [26], [33], [34]. The cross-correlation operation used here is modified from the model of Fischer et al. [34] to include a gain control mechanism. We describe the modified cross-correlation in two stages. The first stage corresponds to the processing of sounds by the cochlear nucleus magnocellularis (NM); this stage represents the input to the cross-correlation operator. The second stage corresponds to the coincidence detectors in nucleus laminaris (NL) where ITD is computed [35].

The input to the cross-correlation in each frequency channel is a gain-modulated version of the filterbank output [33]. The gain at each frequency is a function of the energy, designed so that the magnitude of the filterbank output is a linear function of stimulus level (Figure S1), consistent with the population responses of auditory nerve fibers in the owl [36]. The energy is computed over a short time window by squaring and temporally smoothing the filterbank output. We define the energy mathematically as 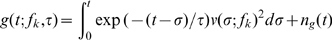, where 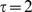 ms. The input to the cross-correlation on one side of the brain is formed by normalizing the filterbank output with a function of the energy, yielding 
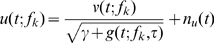
, where 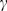 = 100 is a constant that ensures that the denominator is nonzero.

In the barn owl, cross-correlation is performed over a short time window [37] and includes a mechanism that reduces the sensitivity of coincidence detectors to ILD [38]. We model this process of ITD computation by a gain-modulated running cross-correlation, defined by 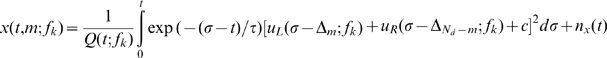 where 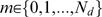 is the delay index, 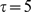 ms is the time constant for the window of integration [37], 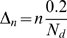 ms is the internal delay on one side of the brain [35], and the constant *c* = 1 causes the input to the squaring nonlinearity to be positive so that responses are consistent with phase-locking in NL by having only one peak per stimulus cycle [34]. The gain 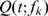 is a quadratic function of the magnitude of the left and right cross-correlation inputs, defined as 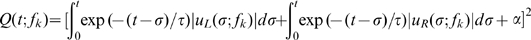, where 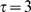 ms and 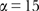 = 15 is a constant that causes the gain term to be nonzero. The gain control on the cross-correlation causes the dependence of the output on stimulus level to be sigmodal and improves the output tolerance to ILD, as is observed experimentally (Figure S1; [38], [39]).

ILD is extracted from the auditory input signals using a level subtraction operation. Specifically, ILD is computed using the interaural difference of a logarithmic function of the energy of the signals in each frequency channel [26], [40], [41]. The logarithmic function of the energy of the gamma-tone filter output employed in the model, hereafter called the energy envelope [40], is defined as
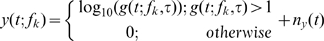
where 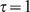 ms. A logarithmic function of the energy 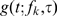 was selected as the envelope because a difference between the left and right envelope signals leads to a roughly stimulus invariant representation of ILD [40]. In particular, the interaural energy envelope difference 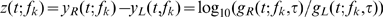 produces a division between the left and right energy terms that removes stimulus effects that are common to the left and right auditory inputs, while preserving information about ILD.

The front end of the model therefore follows the form taken by many sound localization models and includes a bandpass filterbank followed by a cross-correlation and level subtraction operation in each frequency channel [42]. The particular form of the model is, however, tailored to the responses of neurons in the owl's auditory system.

#### 2.2.2 ITD-ILD convergence in ICcl

Previous theoretical studies have shown that multiplicative spiking responses can occur in neurons where input variables are combined additively in subthreshold responses if the input-output function of the neuron follows a power-law [43], [44]. Consistent with this idea, we assume that ITD- and ILD-dependent cues are encoded additively in the membrane potential of ICcl neurons. Specifically, the membrane potential response of the *i^th^* neuron at frequency 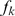 is a low-pass filtered version of the sum of ITD-dependent and ILD-dependent input signals, and is defined by the differential equation
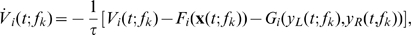
where 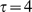 ms and 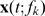 represents the cross-correlation vector across all internal delays. This is the key equation that defines the interaction of ITD and ILD in the subthreshold responses of ICcl neurons. The ITD-dependent input is a scaled version of one element of the cross-correlation vector, 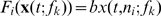, where the identity of the element 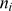 determines the neuron's best ITD. The ILD-dependent input is a product of an ILD-dependent component and energy-dependent component,
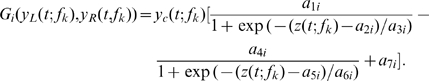
The energy-dependent component is given by the contralateral energy envelope, 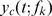, because ICcl receives excitatory input from the contralateral nucleus angularis. We assume that the left and right LLDp contribute only the energy-independent interaural envelope difference [20].

The ILD-dependent component here is a difference of sigmoids; selection of different parameters can produce ILD tuning curves that range from sigmoidal to peaked, as is seen in ICcl [7], [45], [46].

**Figure 1:**
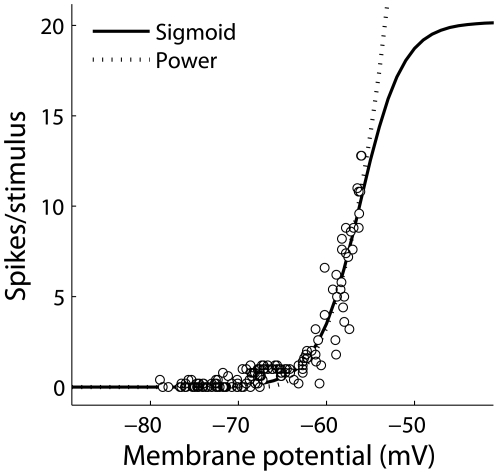
Input-Output response of an ICx neuron. The mapping from the average membrane potential of an ICx neuron over the presentation of a sound stimulus to the number of spikes produced is fit with a power function (dotted line) and a threshold-sigmoid function (solid line). The threshold-sigmoid function is used as the input-output nonlinearity for model ICcl and ICx neurons.

The membrane potential 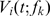 is mapped to a spiking probability using a threshold-sigmoid nonlinearity, rather than the power-law nonlinearity suggested by Murphy and Miller [43]. While no data are available to constrain the input-output properties of ICcl neurons, the threshold-sigmoid nonlinearity provides a good fit to the expansive input-output properties of ICx neurons (Figure 1) [47]. In contrast to the power-law nonlinearity, the threshold-sigmoid nonlinearity allows for the spiking response to saturate, which occurs in ICcl spiking responses [7]. Nevertheless, as seen in Figure 1, the threshold-sigmoid nonlinearity closely matches a power-law nonlinearity over the range of the data for an example ICx neuron, and we therefore should expect this nonlinearity to produce multiplicative spiking responses in ICcl [43], [44]. With this model, the spiking probability for an ICcl neuron is given by the piecewise defined function
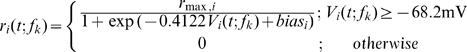
where *r_max,i_* is the maximum firing rate and *bias_i_* determines where the center of the dynamic range of the membrane potential response falls relative to the fixed spiking threshold of −68.2 mV. Both of these parameters are neuron-specific. The fixed parameters are found by fitting the curve to the measured ICx data shown in Figure 1. We define the difference between the threshold value of −68.2 mV and the center of the dynamic range of the neuron's membrane potential response to ITD and ILD as Δ_threshold_. Spikes are produced using a non-homogeneous Poisson process with underlying rate 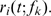 This model for ICcl neurons is the commonly used linear-nonlinear-Poisson model [48].

#### 2.2.3 Frequency convergence in ICx

Spikes are produced for each ICx neuron using a linear-nonlinear-Poisson model. ICx neurons have broad frequency tuning [27], and are therefore considered as a single population where frequency channels are merged. The membrane potential of the 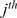 ICx neuron is given by a linear combination of filtered spikes of ICcl neurons across frequency channels,
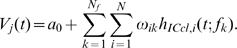
In the simulations, we used 300 total ICcl neurons across two to five frequency channels as input to a given ICx neuron. Postsynaptic potentials in the target ICx neuron are modeled by a second-order exponential function, and responses to multiple spikes are combined linearly. Mathematically, the input to an ICx neuron from the 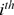 ICcl neuron at frequency 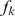 is given by 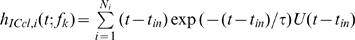 where 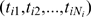 are spike times for the 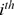 ICcl neuron and 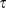 = 4 ms.

The two central properties of this ICx model are, first, that frequency integration in the membrane potential is linear [29] and, second, that multiplication between ITD- and ILD-dependent signals occurs only within frequency channels. The incorporation of linear frequency integration in the membrane potential is an important constraint that is imposed by the data [29]. Connection weights between ICcl neurons and the target ICx neuron are selected so that the membrane potential response of the ICx neuron is a product of ITD- and ILD-dependent functions in each frequency channel. First, we suppose that the measured membrane potential response of an ICx neuron to ITD and ILD can be written as a sum over frequency of terms that are a product of an ITD-dependent function 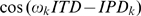 and an ILD-dependent function 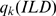, yielding the approximation 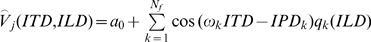. The ILD-dependent function 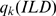 can take an arbitrary value at each ILD. We call this model of the subthreshold ICx response the multiplication-linear model. This model can be directly related to the approximation obtained using a purely multiplicative interaction of ITD- and ILD-dependent components found from the singular value decomposition (SVD) [6]. The parameters of the frequency-specific functions 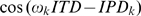 and 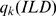 are found by minimizing the squared difference between the measured membrane potential of the ICx neuron and the approximation, averaged over ITD and ILD, using the Matlab function lsqnonlin. Next, the connection weights in each frequency channel of the spiking model 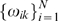 are found to minimize 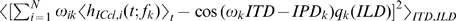, i.e., the squared difference between a linear combination of the time average of the filtered ICcl spikes at frequency 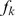 and the desired product of ITD- and ILD-dependent functions at frequency 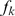, averaged over ITD and ILD. It is reasonable to compute the weights separately for each frequency channel, given the experimental evidence that learning in the owl can elicit frequency-dependent connectivity changes in ICx [49].

### 2.3 Static Model

We also considered simple static models of ICx responses to ITD and ILD in order to compare the responses of ICx neurons under the assumption of either additive or multiplicative frequency integration. This analysis is complementary to that involving the spiking model presented in the previous section.

In the static model, the stimulus is completely specified by the ITD, ILD, and average binaural intensity (ABI) at an array of frequencies. The vectors of ITD, ILD, and ABI are denoted by *ITD*, *ILD*, and *A*, respectively. For both additive and multiplicative models, we assume that the response of a neuron is determined by its preferred ITD and ILD spectra, denoted *bITD* and *bILD*. Each neuron's best ITD and ILD spectra are given by the ITD and ILD spectra derived from barn owl head-related transfer functions at a given direction [15].

In both the additive and multiplicative models, the response to ITD and ILD at each frequency is given by the product of a Gaussian-shaped function of ILD and a circular Gaussian-shaped function of ITD. Under the additive model, the response of the ICx neuron is
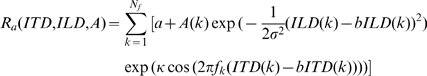
and under the multiplicative model, the response of the ICx neuron is
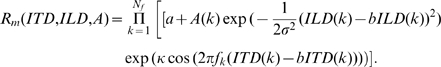
The constant *a* = max(*A*)/200 is included so that the absence of energy in a single frequency channel does not eliminate the response.

These static models are not directly fit to data, but are used to illustrate the different predictions generated under the assumptions of linear and multiplicative frequency combination.

### 2.4 Analysis

We used additional analysis to determine the degree of nonlinearity in the interaction between ITD and ILD. Following previous studies [6], [7], potential nonlinearities were quantified using two types of statistical tests, a linear and a nonlinear fit for each cell.

In the additive fit, the matrix of responses to pairs of stimulus ITD and ILD, denoted *R*, is approximated as the minimum mean square model of the form
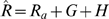
where 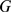 is a function of ITD, 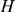 is a function of ILD, and 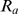 is a constant [6].

The multiplicative fit is specified in terms of the SVD of the response matrix, after the subtraction of a constant bias [6]. A multiplicative fit of the response matrix is obtained using the first singular vectors 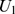 and 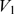 weighted by the first singular value 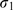 added to a constant bias 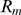, yielding
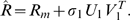



The constant 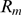 is the value between the minimum and maximum response that minimizes the mean square error in the fit to the data and is distinct from the constant 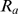 used in the additive fit.

The accuracy of the additive and multiplicative fits is given by the root mean squared (RMS) error between the response and the model fit, normalized by the dynamic range of the neuron's response. The dynamic range is the difference between the maximum and minimum of the response. The relative accuracy of the additive and multiplicative fits is summarized by a multiplication index defined as MI = (nRMS_mult_ − nRMS_add_)/(nRMS_mult_ + nRMS_add_), where nRMS_add_ is the normalized RMS error between the response and the additive fit and nRMS_mult_ is the normalized RMS error between the response and the multiplicative fit.

## Results

**Figure 2:**
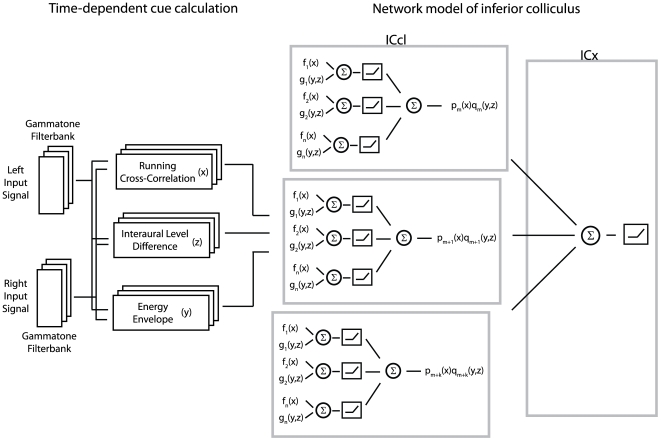
Block diagram of model. The initial components of the model extract time-dependent localization cues using a running cross-correlation, denoted *x*, and the interaural level difference, denoted *z*, from auditory input signals. A network model of spiking neurons uses these cues, along with a measure of stimulus intensity given by an energy envelope (*y*), as input to neurons in the lateral shell of the central nucleus of the inferior colliculus (ICcl), which converge on the external nucleus of the inferior colliculus (ICx). ICcl neurons add a function of the running cross-correlation with another function of the interaural level difference and energy envelope and pass the result through a spiking nonlinearity to produce the probability of spiking. The two central assumptions of the ICx model are, first, that frequency integration at the subthreshold level is linear [29] and, second, that multiplication between ITD- and ILD-dependent signals occurs only within frequency channels. Connection weights between ICcl neurons and the target ICx neuron are selected to enforce these assumptions.

We present a model for the emergence of auditory spatial tuning in ITD- and ILD-sensitive neurons of the barn owl's ICx (Figure 2). ITD- and ILD-dependent cues are extracted from auditory input signals at an array of frequencies using cross-correlation and level-subtraction, respectively. The ITD- and ILD-dependent cues form the input to a network of spiking neurons that model two regions of the inferior colliculus where ITD- and ILD-sensitive neurons are found, ICx and its afferent neurons in ICcl. We assume that the nonlinear spiking responses to ITD and ILD found in ICcl [7] are produced by a nonlinear-additive mechanism [43], [44]. That is, we assume that in ICcl, ITD- and ILD-dependent cues within frequency channels are combined additively in the membrane potential and are transformed to a nonlinear spiking response by the spiking input-output function. The subthreshold responses of ICx neurons can then be described as a sum across frequency of products of ITD- and ILD-dependent functions. The network model describes how a linear combination of the responses of populations of ICcl neurons can compute the products of ITD- and ILD-dependent functions, and thereby produces the experimentally observed responses to ITD and ILD in ICx.

### 3.1 Generating ICcl Responses

#### 3.1.1 Reproducing experimentally measured responses

**Figure 3:**
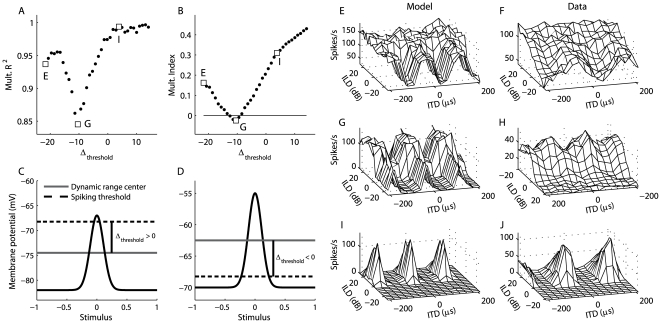
Accuracy of the multiplicative model as a function of spiking threshold in ICcl. The relative accuracy of the additive and multiplicative models of ITD-ILD interaction, summarized by the correlation between the multiplicative fit and the response (A) and the multiplication index (B), depends systematically on the difference between the threshold value of the input-output curve and the center of the dynamic range of the neuron's membrane potential response to ITD and ILD, denoted as Δ_threshold_. Δ_threshold_ is illustrated for positive (C) and negative (D) values. (E,G,I) ITD-ILD response matrices of model ICcl neurons with different thresholds. (F,H,J) Experimentally measured ITD-ILD response matrices [7].

The model was able to reproduce the diversity of combination selectivity observed in ICcl (Figure 3). In the barn owl's ICcl, spiking responses to ITD-ILD range from highly combination selective to more additive than multiplicative [7]. The model produced responses to ITD and ILD that were limited to discrete regions of ITD and ILD space, and were therefore well described by a multiplicative model (Figure 3G). The model also produced responses that were better described by addition than by multiplication (Figure 3E).

The degree of multiplicative interaction of ITD- and ILD-dependent signals in model spike count responses varied systematically with the difference between the spiking threshold and the center of the dynamic range of the subthreshold response of ICcl neurons (Figure 3A,B). We define the difference between the threshold value of the input-output curve and the center of the dynamic range of the neuron's membrane potential response to ITD and ILD as Δ_threshold_ (Figure 3C,D). The types of responses produced by the model as Δ_threshold_ varied (Figure 3E,G,I) are similar to those observed experimentally [7] (Figure 3F,H,J). The variation of the accuracy of a multiplicative fit of the response with Δ_threshold_ can be understood by considering where the dynamic range of the membrane potential response falls on the sigmoidal input-output curve for different Δ_threshold_ values (see section 2.4 for description of additive and multiplicative fits). A low Δ_threshold_ value places the membrane potential dynamic range near the saturation point, a middle Δ_threshold_ value places the membrane potential dynamic range in the linear portion of the curve, and a high Δ_threshold_ places the membrane potential dynamic range near the expansive portion of the curve. Overall, neurons with the highest Δ_threshold_ values had the most multiplicative responses. However, the multiplication index did not vary monotonically with Δ_threshold_. The multiplication index is a measure of the relative accuracy of additive and multiplicative models of ITD-ILD interaction. Over a range of low Δ_threshold_ values, the spiking response was better described by a multiplicative fit than by an additive fit, but the accuracy of the multiplicative fit decreased as the Δ_threshold_ increased (Figure 3A,B). This situation arises because at the lowest Δ_threshold_ values (Figure 3E), the saturation produced by the sigmoidal nonlinearity cannot be captured by the additive fit. As the Δ_threshold_ increases, the dynamic range of the membrane potential falls in the linear portion of the input-output curve, and the spiking response more closely reflects the additive subthreshold response (Figure 3G). For even higher Δ_threshold_, the spiking nonlinearity limits responses to discrete regions of ITD-ILD, causing the spiking response to appear more multiplicative (Figure 3I).

**Figure 4:**
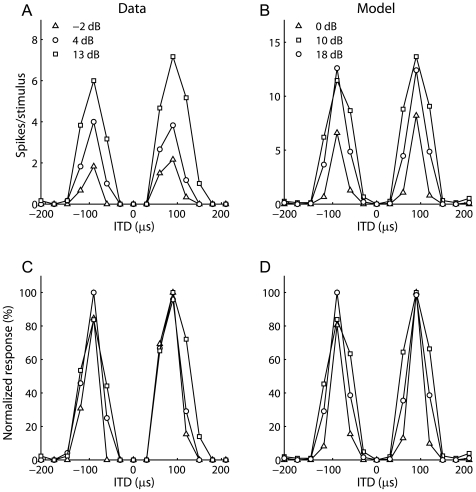
Spiking responses of ICcl neurons are not purely multiplicative. ITD curves obtained at different ILD values were not purely gain-modulated versions of each other in both the experimentally measured responses (A) and the model responses (B). (C,D) Normalized versions of the ITD curves shown in (A,B).

While the model responses are well-described by multiplication, the responses are not purely multiplicative. In particular, the ITD tuning curves for different ILD values are not gain-modulated versions of each other (Figure 4B,D). This is evident, for example, in the variation of the threshold ITD with ILD, which is also seen in the data [7] (Figure 4A,C). However, responses are restricted to particular regions of ITD and ILD, thus acting as an AND gate. Therefore, a multiplicative fit accurately describes the response. Below, we examine how a population of such neurons can combine to produce the experimentally measured responses of ICx neurons.

#### 3.1.2 Predictions for the emergence of multiplicative responses in ICcl

**Figure 5:**
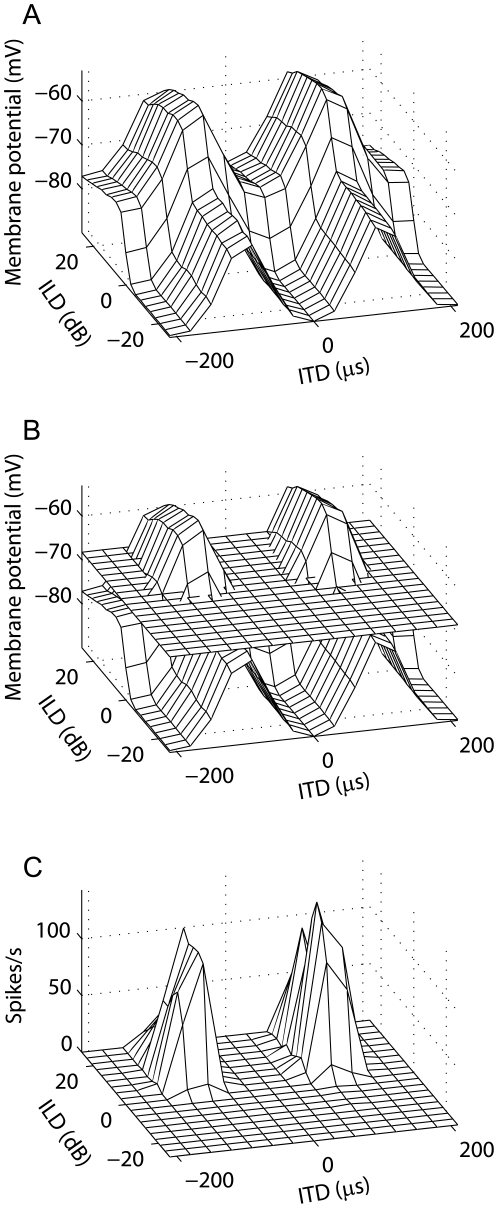
ICcl spiking responses are more multiplicative than are subthreshold responses. The subthreshold response of a model ICcl neuron (A) reflects the additive interaction of ITD and ILD specified in the model form. The spiking threshold (B) limits responses to discrete regions of ITD and ILD, which produces a spiking response that is well described by a multiplicative model (C).

The model predicts that ICcl responses are more multiplicative in spike count responses than in membrane potential responses. By construction, the membrane potential response of the ICcl model neuron is a linear combination of ITD- and ILD-dependent functions. This is evident in that the model shows an increased response when either ITD or ILD is in the preferred range (Figure 5A). As discussed above, the threshold produces a multiplicative spiking response (Figure 5B,C). This prediction can be tested by recording intracellularly the ITD-ILD responses of ICcl neurons.

### 3.2 Generating ICx Responses from ICcl Inputs

#### 3.2.1 Reproduction of experimentally measured ICx subthreshold responses

**Figure 6:**
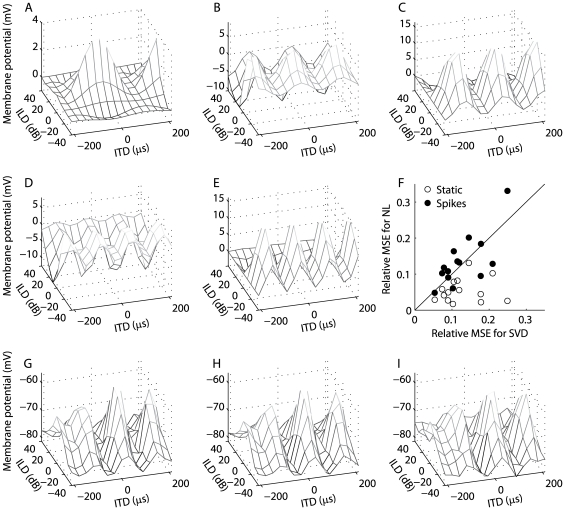
Reproduction of subthreshold responses to ITD and ILD in ICx. The model assumes that ICx subthreshold responses consist of a sum across frequency of products of ITD- and ILD-dependent components. (A–E) Example frequency components for an ICx neuron. (F) Comparison of the relative mean-square-error of the nonlinear-linear model to the relative mean-square-error of the SVD model of responses of 14 ICx neurons. The error is shown for the static and spiking versions of the nonlinear-linear model. The relative error is the mean-square error divided by the dynamic range of the neuron's response. The static (H) and spiking (G) models were able to reproduce the subthreshold responses of ICx neurons to ITD and ILD (I) [6].

Experimentally observed membrane potential responses to ITD and ILD could be produced in model ICx neurons using a linear combination of ICcl spiking responses (Figure 6). We propose that the ICx membrane potential response is a linear combination of products of ITD and ILD-dependent signals at each frequency (Figure 6A–E). This multiplication-linear model provided a more accurate description of the experimentally measured responses than did the purely multiplicative model derived from a singular value decomposition of the response matrix (SVD, median nRMSE 0.11, interquartile range 0.09–0.18; multiplication-linear, median nRMSE 0.05, interquartile range 0.03–0.08; *n* = 14) (Figure 6F; [6]). In the spiking model, the ITD- and ILD-dependent products at each frequency are computed using a linear combination of the responses of a population of ICcl neurons with the same characteristic frequency. This computation is an example of a hidden layer (or basis function) network performing a nonlinear operation on its inputs, and is made possible by the nonlinear input-output function of the ICcl neurons [50]–[52]. We note that each ICx neuron is combining the responses of many ICcl neurons with diverse responses, and is not only selecting the most multiplicative cells. We found connection weights between model ICcl neurons and model ICx neurons to reproduce subthreshold ITD-ILD response matrices for 14 ICx neurons recorded by Peña and Konishi [6] using broadband noise stimuli with frequency-independent ITD and ILD. We tested the ability of the model ICx neurons to reproduce the experimentally measured ITD-ILD response matrices using stimuli not used in the calculation of the connection weights. The normalized root mean squared error between the spiking model response and the measured ITD-ILD response was near the normalized root mean squared error in the SVD model (median nRMSE 0.12, interquartile range 0.09–0.16; *n* = 14). Thus, there is near equivalent performance of the spiking and SVD-based models even though the former is the result of combining populations of Poisson responses and the latter is a direct fit of the data.

**Figure 7:**
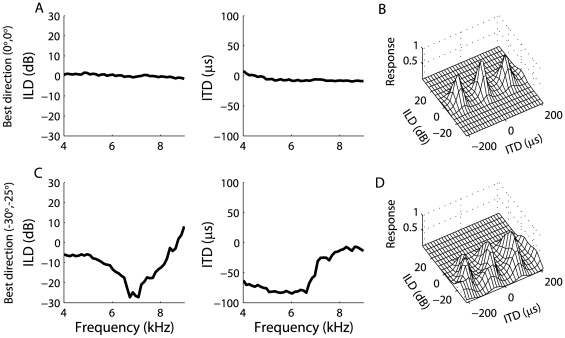
The accuracy of the multiplicative model decreases for ICx neurons with receptive field away from the center of gaze. (A) The ITD and ILD spectra at direction (0°,0°) are approximately constant across frequency [15]. A model neuron with additive frequency integration and best direction (0°,0°) has a response to ITD and ILD that is well described by multiplication (B). (C) The ITD and ILD spectra at directions away from the center of gaze can vary significantly with frequency. (D) A model neuron with additive frequency integration and best direction (30°,−25°) has a response to ITD and ILD that is not as well described by multiplication as the neuron in (B).

Peña and Konishi [6] initially observed multiplicative subthreshold responses of ICx neurons using stimuli presented over headphones with frequency-independent ITD and ILD. The model presented here assumes that frequency integration is linear in the subthreshold responses of ICx neurons and that multiplication only occurs within frequency channels. Therefore, the response is not purely a product of an ITD-dependent component and an ILD-dependent component. How does the multiplicative model derived from the SVD with a single ITD-dependent component and a single ILD-dependent component fit the ICx data so well if the true underlying mechanism involves linear frequency convergence? For directions near the center of gaze, the associated ITD and ILD spectra are relatively constant across frequency (Figure 7A) [14], [15]. If we assume that each neurons' preferred ITD and ILD spectra match the ITD and ILD spectra derived from the HRTFs at the best direction [49], [53], [54], then the inputs to the neuron are very similar across frequency. Therefore, a sum across frequency is approximately equal to a scaled version of a shape that is a product of an ITD-dependent component and an ILD-dependent component. For example, Figure 7B shows the ITD-ILD response of the static model with additive frequency convergence for a neuron with best direction (0°,0°) (see section 2.3 for model description). The squared correlation coefficient between the model response and the SVD-based multiplicative fit is 0.99.

Under natural listening conditions with broadband stimuli, the sounds received at the left and right ears contain a spectrum of ITDs and ILDs [13]–[15]. For a neuron with a preferred direction away from the center of gaze, the preferred ITD and ILD spectra can vary greatly with frequency (Figure 7C). The model we propose for the ICx response predicts that for such neurons, the subthreshold ITD-ILD response matrix will not be accurately described by the multiplicative model derived from the SVD (Figure 7D). Figure 7D shows the ITD-ILD response of the additive static model for a neuron with best direction (−30°,−25°). Here, the squared correlation coefficient between the response and the SVD-based multiplicative fit is 0.85. The only difference between this model response and a model response that yields a correlation of 0.99 (Figure 7B) is the neuron's preferred direction. The deviation from multiplication for neurons with peripherally located receptive fields remains a prediction because of the difficulty in recording ICx neurons with preferred directions away from the center of gaze.

#### 3.2.2 Frequency convergence in ICx spiking responses

**Figure 8:**
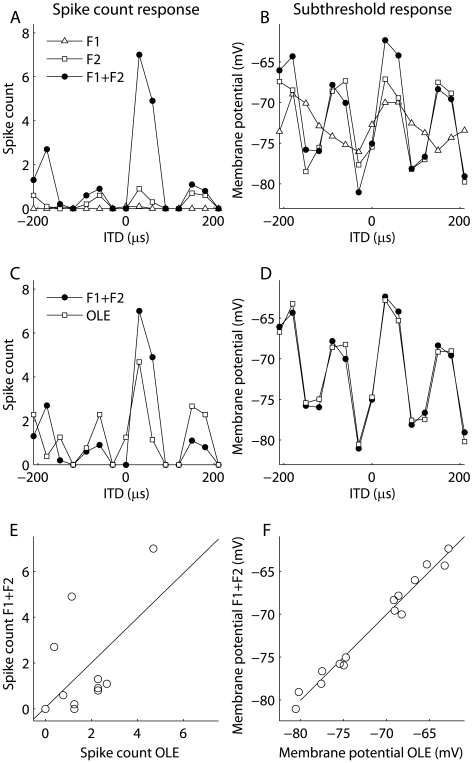
Nonlinear frequency integration in ICx spiking responses. Spiking (A) and subthreshold membrane potential (B) responses to single tones (F1 and F2) and sums of the individual tones (F1 + F2) in a model ICx neuron. (C,D) Approximation of the response to the sum of tones by an optimal linear combination of the responses to the individual tones. (E,F) Comparison of the optimal linear estimate with the response. The solid line is the identity line.

The ICx model produced nonlinear spiking responses to multi-tone stimuli (Figure 8A,C,E). Nonlinear frequency convergence is observed in spiking responses in both ICx and the optic tectum in the barn owl [14], [27], [28]. The model presented here is based on data that show frequency convergence is linear in the membrane potential [29], but also produces nonlinear spiking responses. When the model is tested with stimuli that consist of a sum of two tones, the subthreshold response is well described by a linear combination of the responses to the component tones (Figure 8B,D,F). The spiking response to the two-tone stimulus, in contrast, is not well described by a linear combination of the responses to the component tones (Figure 8A,C,E). Also, for the example in Figure 8, the spiking response to the two-tone stimulus was larger than the optimal linear estimate from the responses to the component tones at some ITDs and smaller than the optimal linear estimate at others. This is consistent with the mixture of facilitation and suppression that is seen in the owl's ICx [28].

**Figure 9:**
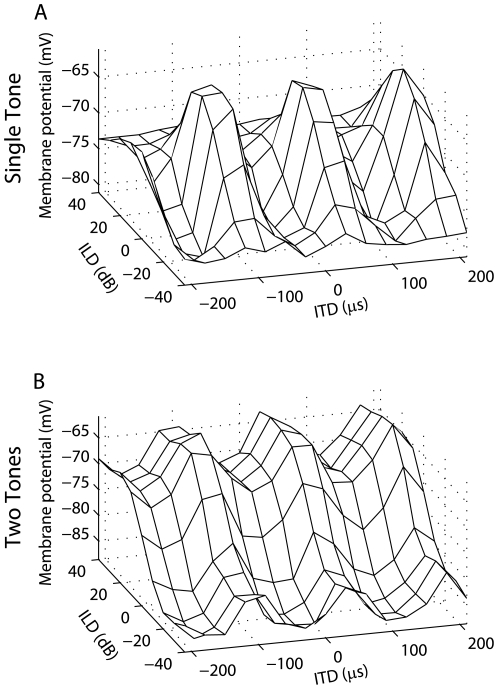
Model ICx responses are more multiplicative for tones than for two tones. (A) Subthreshold ITD-ILD response matrix for a model ICx neuron obtained using a tonal stimulus. Since the model assumes that the response at each frequency is a product of an ITD-dependent function and an ILD-dependent function, the tonal response matrix is accurately described by multiplication. (B) In contrast, the response of the same model neuron obtained when ITD varies in one frequency and ILD varies in a second frequency is not accurately described by multiplication because the model assumes that frequency integration is linear.

This model also leads to the prediction that, in ICx, subthreshold responses to narrowband sounds are more multiplicative than responses to broadband sounds. For example, according to the model, the subthreshold response of an ICx neuron to ITD and ILD of a tone should appear more multiplicative than the response to a sound consisting of two frequency components, where ITD is varied in one frequency channel and ILD is varied in the second (Figure 9). In the example shown in Figure 9, the multiplication index was −0.16 for the one-tone example and 0.37 for the two-tone example. This result follows from the model for the ICx subthreshold response, which specifies that nonlinearity between ITD and ILD only occurs in frequency channels. This prediction is therefore not dependent on the particular ITD and ILD tuning parameters of the neuron. Note that under a purely multiplicative model of frequency integration, the response to either of these conditions would be described as equally multiplicative.

#### 3.2.3 Implications for representing multiple sound sources

**Figure 10:**
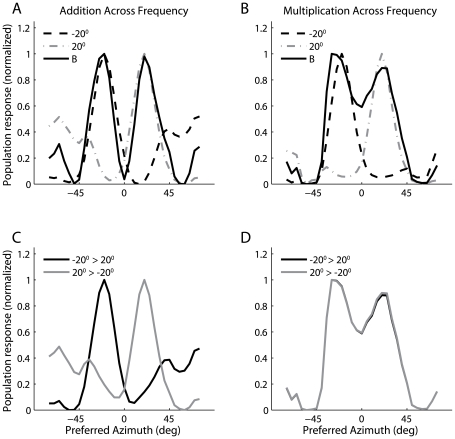
Comparison of the population representation of multiple sound sources for models with additive and multiplicative frequency integration. (A,B) The population response to two simultaneous spectrally distinct sources (solid line) located at −20 deg and 20 deg under the assumption of additive (A) and multiplicative (B) frequency integration. The dotted lines show the responses to the sounds presented alone. (C,D) The population response to two simultaneous spectrally distinct sources when one source is five times more intense than the other. In each plot, the responses are normalized by subtracting away the minimum value and then dividing by the maximum value.

We hypothesize that non-multiplicative frequency integration facilitates the representation of multiple, spectrally distinct sources in the auditory space map. Listening in natural environments requires the localization of multiple distinct sound sources. Neurophysiological studies show that spectrally distinct sound sources create distinct patterns of activity in the auditory space map [30]. Also, when two sound sources are present, the response reflects the more intense sound source [14], [26]. We examined the representation of two spectrally distinct sound sources, located at ±20 deg, in the auditory space map using the static linear and multiplicative models of frequency integration. By construction, with a linear model of frequency integration, the population response to two spectrally distinct sound sources is the sum of the responses to the individual sounds. Therefore, with the linear model, the space map contains peaks at the directions of the two sources (Figure 10A). With multiplicative frequency integration, the response to two sounds that are separated by 40 deg has two peaks, but the peaks are not centered precisely at the directions of the individual sound sources (Figure 10B). Additionally, the trough separating the two peaks is shallower in the multiplicative model than it is in the additive model. A more significant difference appears between the additive and multiplicative models when we examine the population response as the intensities of the two sounds are varied. When one sound is more intense than the other, the additive model produces a response that has a dominant peak at the direction of the more intense source (Figure 10C). The response of the multiplicative model, in contrast, does not change greatly as either sound becomes more intense (Figure 10D). In the multiplicative model of frequency integration, contributions of individual frequency channels to the final product cannot be determined. The response of the multiplicative model of frequency integration model is thus inconsistent with the responses of ICx and optic tectum neurons to concurrent sound sources [14], [26].

**Figure 11:**
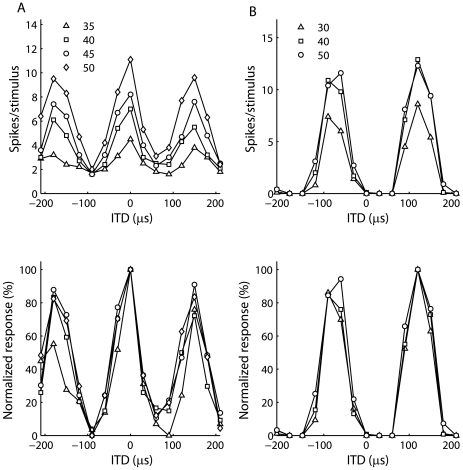
ITD curves at different average binaural levels. ITD curves at different average binaural levels given in spikes/stimulus (top row) and normalized units (bottom row) for two experimentally measured ICcl neurons.

In our linear model of frequency integration, simultaneous, spectrally distinct sounds can be represented in the space map in such a way that the more intense source dominates the response. This means that information about the individual frequency components of the sound is not lost in the responses of spatially selective auditory neurons. This cannot occur with a purely multiplicative model of frequency convergence, where stimulus intensity only modulates the overall gain. However, similar results to the linear model would be obtained with a sum and square model that includes linear and nonlinear frequency integration [26]. Another possible way to represent multiple sources in a multiplicative model is to change the width of the input tuning curves with changes in intensity. If more intense sounds produce sharper tuning curves, then the largest peak in the space map should occur at the direction of the more intense sound. To test this hypothesis, we measured ITD tuning curves of ICcl neurons in three owls for different stimulus intensities. The ITD tuning curves of ICcl neurons did not sharpen greatly with increasing intensity (Figure 11). The half-width of the ITD tuning curve was on average 10.2±27.7 µs smaller for a stimulus intensity of 50 dB than for an intensity 30–35 dB (*n* = 9). In three of nine neurons, the half-width increased with stimulus intensity.

## Discussion

### 4.1 Processing of Sound Localization Cues in the Owl's Midbrain

We described a model for the processing of sound localization cues in the barn owl's midbrain. In this model, ITD- and ILD-dependent signals are combined multiplicatively within frequency channels, and signals are combined across frequency channels using a linear-threshold operation. The model is consistent with neurophyiological studies of both ITD-ILD coding and the representation of multiple sound sources in the owl's midbrain.

The model presented here better captures the ICcl spiking responses and ICx subthreshold responses to sounds than previous models. As in previous models [14], [24]–[26], ours assumes that ITD- and ILD-dependent signals must be combined over multiple frequencies to explain the neurophysiological and behavioral observations. However, the present model provides an extension of previous models on three counts. First, our model produces the diversity of spiking responses to ITD and ILD that are seen in ICcl [7]. This extends previous models by providing a possible mechanism for the generation of nonlinear responses to ITD and ILD in ICcl, and by addressing the issue of how a heterogeneous population of ICcl responses is processed to produce ICx responses. Second, our model examines the subthreshold responses of ICx neurons [6], [29]. This is the first model to reconcile the observation of multiplicative subthreshold responses to ITD and ILD [6] with the presence of linear frequency integration in subthreshold ICx responses [29]. Third, by including an explicit representation of the subthreshold and spiking responses of ICcl and ICx neurons, our model assigns each operation performed on the ITD- and ILD-dependent input signals to a particular processing stage in the inferior colliculus.

This model, along with previous ones, leaves several issues open. First, how are the connections between ICcl and ICx neurons learned? There are several proposals for the mechanisms underlying learning in the owl's auditory system [55]–[62], but none has compared model responses to ITD and ILD over frequency with experimentally measured responses. Second, what dendritic mechanisms are involved in generating responses to ITD and ILD in the inferior colliculus? Models of the owl's inferior colliculus have employed point neurons that neglect processing in dendritic trees. Segregation of ICcl inputs on different branches of the dendritic tree may be important for generating nonlinear responses within frequency channels, while maintaining linear frequency integration [63]. Third, how is the diversity of responses to ITD and ILD seen in ICcl generated from presynaptic inputs? Models should include representations of both the level- and time-pathway inputs to ICcl in order to reproduce this diversity.

### 4.2 Mechanisms for Multiplicative Responses

We propose that the population of neurons in each frequency channel of ICcl acts as the layer that performs multiplication of ITD- and ILD-dependent signals. This model makes simple biophysical assumptions: subthreshold integration of signals is linear, and nonlinearity between ITD and ILD is introduced by the spiking input-output function. Under this model, the responses to ITD and ILD in ICcl are only approximately multiplicative, which is consistent with the data [7]. Thus, a clean multiplication between signals within frequency channels emerges in the subthreshold responses of ICx neurons as a network property. This is an example of a hidden layer (or basis function) network performing a nonlinear operation on its inputs [50]–[52]. ICx responses are produced in the model by a linear feedforward projection of ICcl responses where frequency-specific weights lead to clean multiplicative responses within frequency channels. One advantage of this network is that it can learn sets of frequency-specific weights from ICcl to ICx. This is an important constraint that the network must fulfill, given the experimental evidence for the owl learning frequency-dependent ITD selectivity in ICx [49].

There are several previously proposed models for the generation of multiplicative neural responses. These models show that the mechanism that produces multiplicative responses may be the property of a single cell [43], [44], [64], a recurrent network [65], or a feedforward network [52], [66], [67]. Our model employs both cellular and network mechanisms to produce multiplicative responses. At the cellular level, our model is consistent with previous studies that show that the spiking nonlinearity can produce multiplicative responses [43], [44], [64]. Mel [64] showed that adding and rectifying two peaked functions produces a response that is very similar to the product of the two peaked functions. Murphy and Miller [43] found that multiplicative responses could be produced by neurons that sum their inputs if the spiking nonlinearity follows a power-law. Our model of ICcl is consistent with these cellular models; the input to model ICcl neurons is a sum of peaked functions of ITD and ILD, and spiking responses are a threshold-sigmoid function of the input. The threshold-sigmoid function matches a power-law function over a large portion of its dynamic range. This mechanism is able to produce responses that are well described by multiplication. By varying where the spiking threshold falls on the dynamic range of the subthreshold response, the model also produced responses that were not very multiplicative, as is seen in the data [7]. Previous studies have also shown that products of variables can be read out from the responses of a population of neurons with a diversity of nonlinear responses [52], [67]; this is the hidden layer model for performing multiplication. In our model, the population of neurons in each frequency channel of ICcl acts as the hidden layer for performing multiplication of ITD- and ILD-dependent signals. In summary, we propose that the owl's inferior colliculus employs a combination of plausible cellular and network mechanisms to produce multiplicative responses.

A study by Poirazi et al. [68] raises the question of why the owl's auditory system employs ICcl as a separate neural hidden layer for performing multiplication when dendritic mechanisms could alternatively be used. Poirazi et al. [68] showed that the dendritic tree of a single neuron can act as a two-layer neural network. Under this model, it should be possible for ICx neurons to use local dendritic processing to introduce a nonlinearity between ITD- and ILD-dependent inputs within frequency channels. One reason that ICcl is used, rather than local dendritic nonlinearities, may be the complexity that dendritic hidden layers would add to learning rules. In the dendritic hidden layer, time- and level-pathway inputs from the same frequency channel must synapse not only on the appropriate neuron, but on a particular segment of the dendritic tree. A second reason for using a neural hidden layer is that the intermediate results, the products within frequency channels, may need to be used by both the midbrain and forebrain [69], [70]. If a dendritic hidden layer is used, then the intermediate results cannot be shared between neurons.

### 4.3 Model Predictions

Based on the model, we make three specific predictions about responses of neurons in the midbrain of the owl to ITD and ILD. First, in ICcl, spiking responses to ITD and ILD will be more multiplicative than subthreshold responses. This prediction is based on our hypothesis that multiplicative responses emerge in ICcl neurons as a result of the spiking nonlinearity. In our model, subthreshold integration of ITD and ILD is purely linear, but this may not be satisfied in the owl because of synaptic nonlinearities that the model does not account for. Nevertheless, we predict that spiking responses will be more multiplicative than will subthreshold responses. This prediction can be tested by recording intracellularly the responses of ICcl neurons to ITD and ILD. Second, in ICx, neurons with preferred directions away from the center of gaze will have less multiplicative subthreshold responses to ITD and ILD than will neurons with preferred directions near the center of gaze. This occurs in our model because addition and multiplication across frequency are clearly distinguished for neurons with highly frequency-dependent preferred ITD and ILD spectra. This prediction can be tested by recording intracellularly the responses of ICx neurons with preferred directions away from the center of gaze to ITD and ILD. Third, we predict that frequency integration is not multiplicative in the spiking responses of neurons in ICx and the optic tectum; the response to two tones should appear as a linear-threshold combination of the responses to the individual tones [29]. Nonlinearity is observed in the frequency integration responses of ICx and optic tectum neurons [14], [27], [28], but we propose that this is due to a threshold-nonlinearity, not to multiplication. While a threshold operation forms a close approximation to multiplication, there is an important distinction between the models. Under the linear-threshold model, the influence of a single frequency component on the neural response varies with the intensity, while under the multiplicative model it does not. The presence of a linear-threshold nonlinearity is supported by current data showing that the more intense of two simultaneous spectrally distinct sources will have the greater influence on the response of these neurons [14], [26] and that frequency integration is linear in subthreshold responses of ICx neurons [29]. This prediction can be further tested by comparing ITD-ILD responses matrices obtained for narrowband sounds and for sounds consisting of two frequency components where ITD is varied for one frequency and ILD varied for the other.

## Supporting Information

Figure S1 (A) Linear output of the gain-modulated gammatone filter (measured as root-mean-square, RMS), as a function of average binaural level of a broadband noise. (B) Cross-correlation vector as a function of average binaural level. (C) Tolerance to ILD of the cross-correlation vector.
(0.17 MB PDF)
