# Supplementary figures and images for: Multiplicative Auditory Spatial Receptive Fields Created by a Hierarchy of Population Codes
Source: PLoS One. 2009 Nov 24;4(11):e8015. doi: 10.1371/journal.pone.0008015 (PMC2776990; doi:10.1371/journal.pone.0008015)

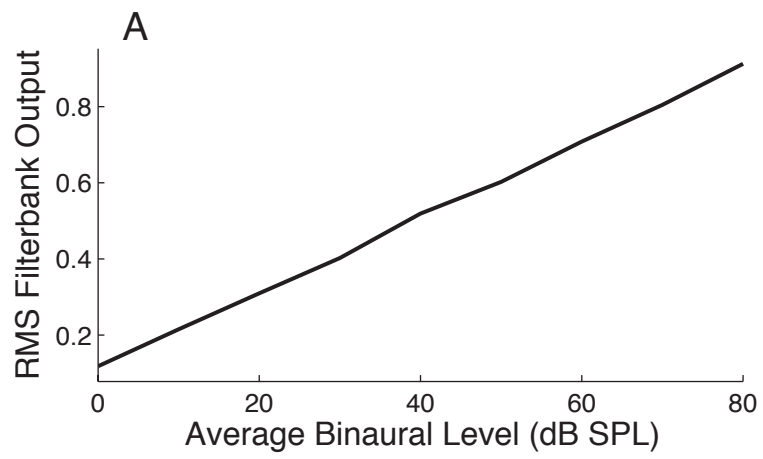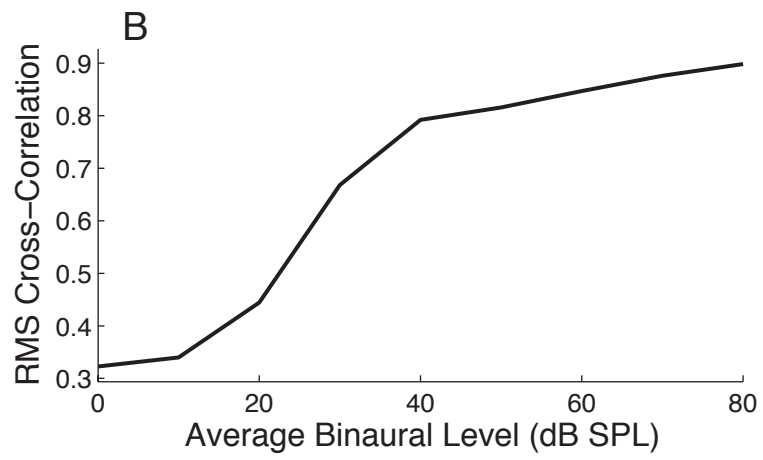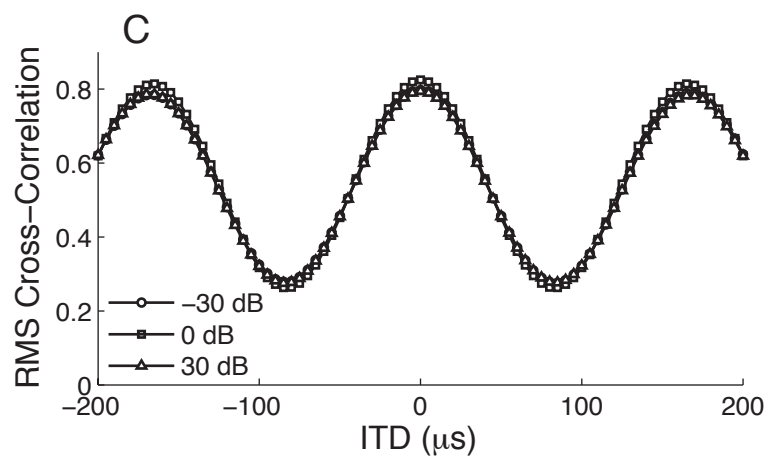

Supplement: Figure S1 — (A) Linear output of the gain-modulated gammatone filter (measured as root-mean-square, RMS), as a function of average binaural level of a broadband noise. (B) Cross-correlation vector as a function of average binaural level. (C) Tolerance to ILD of the cross-correlation vector. (0.17 MB PDF) [file pone.0008015.s001.pdf]
